# A Randomized Controlled Trial for Prevention of Postspinal Anesthesia Shivering in Gynecological Surgeries: Mirtazapine vs. Dexamethasone

**DOI:** 10.1155/2022/5061803

**Published:** 2022-03-09

**Authors:** Ibrahim M. Esmat, Ahmed M. Elsayed, Hazem M. El-Hariri, Tarek M. Ashoor

**Affiliations:** ^1^Department of Anesthesia and Intensive Care, Faculty of Medicine, Ain-Shams University, Cairo, Egypt; ^2^Community Medicine Department, National Research Centre, Cairo, Egypt

## Abstract

**Background:**

The frequency of shivering regarding regional anesthesia is 55%. Newer effective and tolerable options for postspinal anesthesia shivering (PSAS) prophylaxis are necessary to improve patients' quality of care. This research assessed the impact of preemptive mirtazapine versus preemptive dexamethasone to decrease frequency and severity of PSAS in gynecological procedures.

**Methods:**

300 patients booked for gynecological procedures under spinal anesthesia (SA) were randomly apportioned into three groups (100 each) to get one preemptive dose of 30 mg mirtazapine tablet (M group), 8 mg dexamethasone diluted in 100 ml of saline infusion (D group) or placebo (C group) two hours before surgery. Incidence of clinically significant PSAS was the primary outcome. Core temperature, shivering score, hemodynamics changes, adverse events, and patient satisfaction score were documented as secondary outcomes.

**Results:**

Compared with C group, mirtazapine and dexamethasone decreased incidence of clinically significant shivering (74% vs. 16% and 31%, respectively; *P* < 0.001). M and D groups had less hypotensive episodes during 5–25 min after intrathecal injection (*P* < 0.001). 90 min after SA, tympanic temperatures were lower than baseline values in the three groups (*P* < 0.001). Pruritus, nausea, and vomiting were more often in C group (*P* < 0.001), whereas sedation was more frequent in M group (*P* < 0.001). C group had the lowest satisfaction scores (*P* < 0.001).

**Conclusion:**

Prophylactic administration of mirtazapine or dexamethasone attenuated shivering with minimal hazards in patients scheduled for gynecological surgeries under spinal anesthesia with priority to mirtazapine. The trial is registered with NCT03675555.

## 1. Introduction

Spinal anesthesia (SA) is commonly practiced in gynecological surgeries. SA has many advantages, e.g., less intraoperative bleeding, less risk of venous thromboembolism, and better pain relief. Complications may happen, e.g., hypotension, postdural puncture headache (PDPH), and shivering [1]. The frequency of shivering regarding regional anesthesia is 55% [2].

SA influences temperature regulation through promotion of heat loss by vasodilation leading to trunk hypothermia and shivering [3, 4]. Several mechanisms are involved in the pathogenesis of postspinal anesthesia shivering (PSAS) including intraoperative heat loss and endogenous pyrogens. PSAS exerts metabolic effects and hemodynamic impacts including increased oxygen consumption, excess carbon dioxide production, high catecholamine levels in plasma, and increased cardiac output [2]. Patients may feel uncomfortable about the PSAS which may interfere with ECG monitoring, measurements of blood pressure, and readings of O_2_ saturation [4]. Women vary from men in their more prominent subcutaneous fat layer and poor exercise capacity resulting in different thermal reactions to external and internal heat loss during rest and exercise [5]. Nonpharmacological interventions (e.g., warm fluids infusion and forced-air warming devices) provide inadequate control of central hypothermia and hence the need for drugs for both treatment and prophylaxis of shivering [6]. Newer effective and tolerable options for PSAS prophylaxis are necessary to improve patients' quality of care.

The antagonism of the serotonergic system was found to lower hypothalamic temperature set threshold, therefore reducing metabolic cold defense and suggesting a role in postoperative shivering control [7]. Mirtazapine is a noradrenergic and a serotonergic antidepressant (NaSSA). Mirtazapine antagonises central *α*2-auto- and hetero-adrenoreceptors enhancing release of both noradrenergic and 5-HT_1A_-mediated serotonergic neurotransmission [8]. Moreover, mirtazapine has anxiolytic, antinausea, and antiemetic effects due to blocking of 5-HT_2_ and 5-HT_3_ receptors [9]. In addition, mirtazapine has an antinociceptive effect [8] and decreases incidence of PDPH after SA [10]. Mirtazapine is promptly absorbed and its peak plasma concentration (C-max) is available within 1 to 2.1 h [11].

Dexamethasone diminished the frequency of shivering following open-heart surgeries. Anti-inflammatory properties of dexamethasone allow reduction of temperature gradient between tympanic and skin temperatures [12]. Preoperative 8 mg intravenous (IV) dexamethasone improved quality of recovery in patients scheduled for laparoscopic cholecystectomy in comparison to placebo-treated patients [13–15]. IV dexamethasone may cause burning perineal sensation in 50–70% of awake patients [13].

This research assessed the impact of preemptive mirtazapine versus preemptive dexamethasone to decrease the incidence and severity of PSAS in gynecological procedures.

## 2. Methods

### 2.1. Study Population

This study was conducted between March and August, 2018, after approval of the local ethical committee (FMASU R 47/2018) on 300 women, aged 18–60 years and ASA I or II scheduled for elective gynecological surgeries under SA. This study was registered at ClinicalTrials.gov (NCT03675555) and followed the regulations and amendments of the Helsinki Declaration-2013. Every patient who chose to participate in this research signed a consent.

Exclusion criteria were diabetes mellitus, thyroid disease, cardiopulmonary disease, bleeding tendencies, neurologic disease, psychological disorders, liver dysfunction, a body mass index (BMI) >35 kg/m^2^, body temperature <36.5°C or >38.0°C, history of substance abuse, treatment with sedative hypnotic agents, medications altering thermoregulation, vasodilators, allergy to the study medications, and contraindications to SA. Patients were also ruled out if they refused to participate in clinical research, required blood transfusion during procedure, or had operation time >120 min. If patients did not achieve satisfactory bilateral sensory block level or Bromage score 3 motor blockade, they received general anesthesia and were excluded from this research.

All selected patients underwent routine preoperative medical check, preoperative and postoperative hemoglobin concentration analysis, 6 h preoperative fast for solid food, and 2 h preoperative fast for clear fluids.

### 2.2. Randomization and Blinding

Patients were randomized into 3 groups (100 each) in a 1 : 1 : 1 allocation ratio in accordance with shivering prevention protocol using computer-generated random numbers concealed in sealed opaque envelopes, and a nurse randomly chose the envelope to determine the assigned group [16]. Patients were allocated to mirtazapine (M) group, dexamethasone (D) group, or control (C) group and obtained shivering prophylaxis protocol 2 h before surgery. In M group, patients obtained 30 mg mirtazapine tablet with sips of water and an identical-looking placebo 100 ml 0.9% sodium chloride (normal saline [NS]) intravenous infusion (IVI) over 15 minutes. In D group, patients obtained 8 mg/2 ml dexamethasone ampoule mixed with 100 ml 0.9% NS IVI over 15 minutes and an identical-looking placebo tablet, whereas in C group patients obtained an identical-looking placebo tablet and solution.

Intervention drugs, including mirtazapine and dexamethasone, were in the form of Remeron® tablets manufactured by Organon NV/Netherlands and Dexamethasone Sodium Phosphate® 8 mg/2 ml, ampoules, MUP Egypt. The hospital pharmacy was responsible for preparation of the study drugs which were delivered to ward nurses to be given to patients. Follow-up notes were documented by anesthesia residents. Patients, ward nurses, gynecologists, and anesthesia residents were blinded to the patient's group assignment [16].

### 2.3. Study Protocol

The research team applied the same anesthetic management and the same quality of care to all patients involved in this study. Before commencing SA, no premedication was given, standard monitoring was established including tympanic membrane (core) temperature (*T*), and each patient received 10 ml/kg IV Ringer's lactate preload. Core temperature was measured by Braun ThermoScan® IRT 4020 ear thermometer [17]. Operating room temperature was provided in a range of 23–25°C and 60 to 70% relative humidity. Hypothermia was developed if the core temperature dropped below 36.5°C.

Intrathecal block was performed at L3-4 or L4-5 interspace through the midline approach with patient in sitting position using a 25-gauge Quincke spinal needle. The attending anesthesiologist injected 2.5–3.5 ml (12.5–17.5 mg) of 0.5% hyperbaric bupivacaine to reach the desired surgical level taking into consideration patient's height and weight. By the end of SA technique, the patient lied supine, an oxygen face mask was applied at a rate of 5 L/min, covered with a standard single blanket and did not receive any active perioperative warming.

Pinprick test was used to assess the peak sensory level, time to reach this level (min), and time to two-segment regression (min) after the intrathecal bupivacaine administration (starting point of this research). Anesthesia residents reported success of SA if a bilateral T4-T8 sensory block to pinprick test within 15 min of intrathecal drug administration happened and they also documented time to rescue analgesia (min). Motor block was evaluated by using modified Bromage score [18] to determine time to reach maximum motor block (Bromage score 3) (min) and duration of motor block (min).

Hemodynamics of patients including heart rate (HR), mean arterial pressure (MAP), peripheral arterial oxygen saturation (SPO_2_%) and T were documented before intrathecal injection (baseline) and thereafter at 2 and every 5 min till the first 30 minutes after SA and then at 10-minutes intervals till 90 min after SA (ending point of the study).

Shivering severity was assessed by a scale of 4 grades 0; no shivering, 1; mild shivering, 2; moderate shivering, 3; severe shivering [19]. Two anesthesia residents, unaware of the study intervention allocation, documented the grades of shivering till 90 min after the subarachnoid block. If the shivering grade developed to equal or more than 2 (clinically significant PSAS) after 15 min from the completion of SA, the preventive protocol for PSAS was considered inefficient and 25 mg IV meperidine was administered. Onset of shivering, response rate, and shivering recurrence were also reported. Response rate is the complete suspension of shivering activity within 10 min after the first dose of meperidine. Satisfaction of patients with shivering prevention protocol was evaluated with seven-point Likert rating scale [20].

The research team documented any adverse events including hypotension (MAP <20% from prespinal values), bradycardia (HR <50 beats/min), respiratory depression (respiratory rate ≤8/min or oxygen saturation ≤92%), pruritus, nausea, vomiting, headache, and dry mouth. Hypotension was treated with 250 ml crystalloid infusion and/or incremental dose of 6 mg IV ephedrine. If a patient complained of hypotension and nausea at the same time, an incremental dose of 6 mg IV ephedrine was given. 0.01 mg/kg IV atropine was administered if bradycardia occurred. Patients with nausea (>10 min) and/or vomiting (≥2 episodes) were treated with 10 mg IV metoclopramide. Pruritus was managed with 2 mg IV clemastine (Tavegyl®). Sedation was evaluated every 15 min over 90 min after SA and was assessed with a scale of four points as per Filos et al. [21]. The research team members collected blood samples from all patients preoperatively and one week after surgery to compare liver enzymes level (SGPT).

The incidence of clinically significant PSAS occurring during the first 90 min after SA was considered as a primary outcome. Secondary outcomes included evaluation of core temperature, shivering profile, satisfaction of patients with shivering prophylaxis protocol, and adverse events.

## 3. Statistical Analysis

### 3.1. Power of the Study

Based on earlier research, a sample size of 19 cases in every group was required to keep a statistical significance when the expected incidences among the three groups were as follows: group C (33.3%), group P (0.0%), and group D (0.0%) [22] with adjusting *α* = 0.017, *β* = 0.80 [23] and calculating with PASS 11th release [24]. The research team allocated 100 cases for each study group to account for possible attrition and to detect possible adverse effects.

### 3.2. Data Analysis

The gathered data were managed and analyzed using IBM SPSS statistics (Statistical Package for Social Sciences) program 22.0^th^ release, IBM Corp., Chicago, USA, 2013. Quantitative data were described as mean ± SD (standard deviation) and then were compared using ANOVA test and repeated measures analysis of variance (RMANOVA) if normally distributed. If data were not normally distributed, median and 1st & 3rd interquartile range were used for description and Kruskal Wallis test for comparison. While in conditions of qualitative data, number and percentage were used for description and each of chi square test and Fisher's exact test for comparisons depending on expected number size. Rates were compared using Log rank test. *P*- value <0.050 was set as a significance cut point. Bonferroni test was used for post hoc comparisons.

## 4. Results

**Figure 1:**
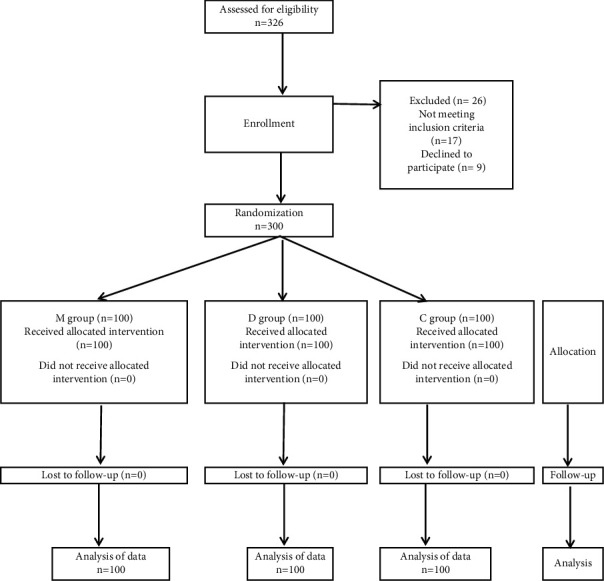
Consort flow chart.

Among the 326 female patients who were screened for eligibility, 300 patients were properly enrolled and subjected to statistical analysis. A consort flow chart is presented in Figure 1. There were no statistically significant differences in demographics or confounders between the 3 groups (Table 1).

More patients in M and C groups reached the peak sensory level in a significantly long period of time compared to D group (*P* < 0.001) (Table 1) with no significant difference between M and C groups. D group patients had a significantly more time for two-segment regression and a significantly more time for rescue analgesia in comparison to M and C groups with significant differences between M and C groups (*P* < 0.001, *P* < 0.001, respectively) (Table 1). There were no significant differences between groups as regards the peak sensory level, the time to reach maximum motor block, and the duration of motor block (*P*=0.389, *P*=0.062, *P*=0.065, respectively) (Table 1).

**Figure 2:**
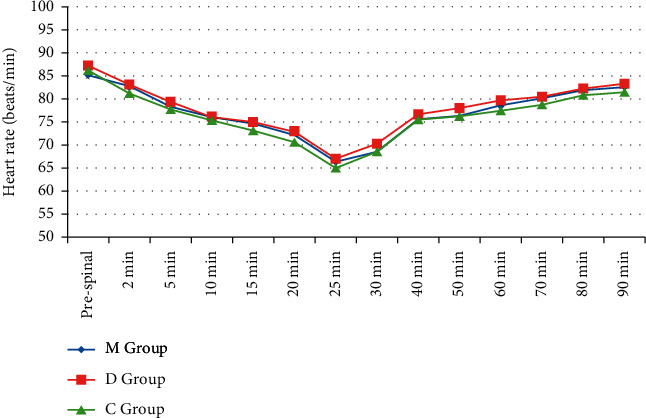
Heart rate (beats/min) changes over 90 minutes among the studied groups.

**Figure 3:**
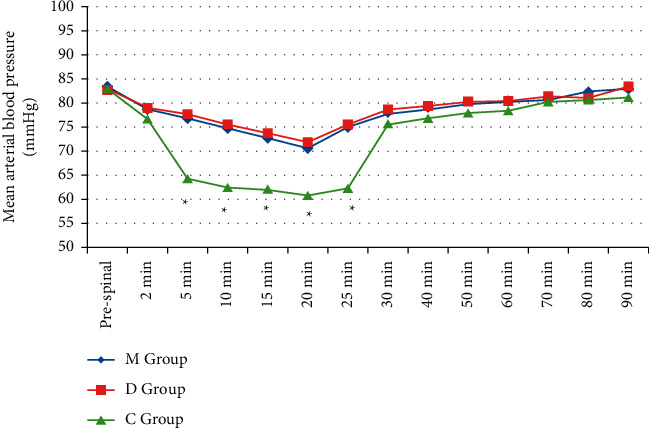
Mean arterial blood pressure (mm·Hg) changes over 90 minutes among the studied groups. ^*∗*^Statistically significant.

Alterations of heart rate were comparable between the three groups till 90 min after SA (*P* > 0.05) (Figure 2). More cases in M and D groups exhibited higher MBP values till 25 min after SA in comparison to C group (*P* < 0.001) with comparable efficacy between M and D groups (Figure 3). Alterations of SpO_2_ (%) were comparable between the three groups till 90 min after SA (*P* > 0.05).

**Figure 4:**
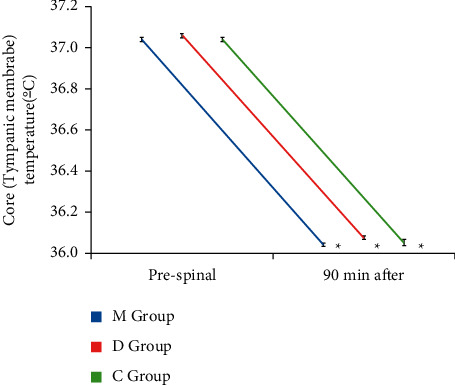
Variations in core temperature (°C) after 90 minutes of subarachnoid block in comparison to the prespinal (baseline) values. ^*∗*^Statistically significant.

Core temperature values 90 min after SA were significantly decreased in the three groups in comparison to baseline values (*P* < 0.001) (Figure 4) without significant difference when compared to each other (*P* > 0.05).

**Figure 5:**
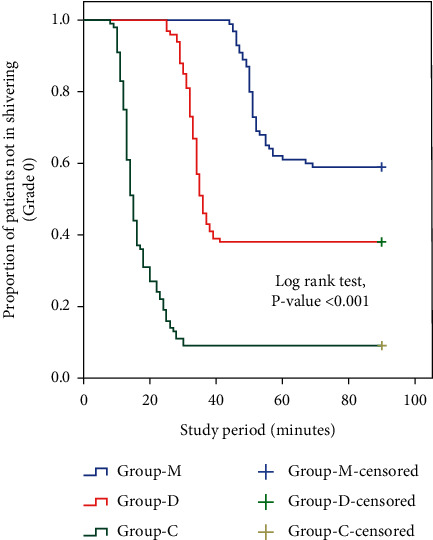
Proportions of patients not in shivering (Grade 0) represented on the Kaplan Meier plot.

**Figure 6:**
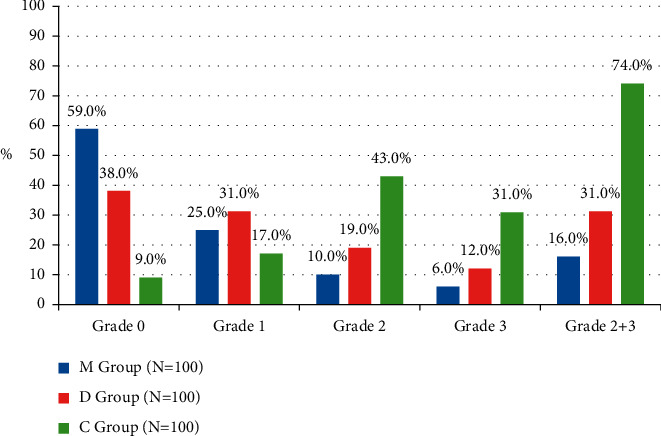
Patients' percentages of different grades of shivering after 90 minutes of subarachnoid block.

In C group, the incidence of shivering was higher whereas the onset of shivering was lower than the other two groups with significant differences between M and D groups (*P* < 0.001, *P* < 0.001, respectively) (Table 2) (Figures 5 and 6). The incidence of clinically significant shivering was higher in C group (74.0%) in comparison to M group (16.0%) and D group (31.0%) with significant differences between M and D groups (*P* < 0.001) (Table 2) (Figure 6). In C group, the mean dose of meperidine was higher whereas the response rate after single dose of meperidine was lower than the other two groups with significant differences between M and D groups (*P* < 0.001, *P*=0.002, respectively) (Table 2). The recurrence of shivering was recorded in 9/31 (29.0%) patients of D group and in 33/74 (44.6%) patients of C group; on the contrary, no recurrence of shivering was documented in M group (*P*=0.002) (Table 2).

In C group, incidence of postspinal anesthesia (PSA) hypotensive episodes, the administered ephedrine, and the need for ephedrine to treat hypotension were more frequent than the other two groups with comparable efficacy between M and D groups (*P* < 0.001, *P* < 0.001, *P* < 0.001 respectively) (Table 3). Incidences of pruritus, nausea, vomiting, and use of rescue antiemetic were higher in C group than the other two groups with comparable efficacy between M and D groups (*P* < 0.001, *P* < 0.001, *P* < 0.001, *P* < 0.001, respectively) (Table 3). In M group, sedation scores and incidence of dry mouth were higher than the other two groups (*P* < 0.001, *P* < 0.001, respectively) (Table 3) with no statistically significant differences between D and C groups. More patients in M and D groups were satisfied with shivering prophylaxis protocol in comparison to C group (*P* < 0.001) (Table 3) with comparable efficacy between M and D groups.

## 5. Discussion

The research team had found that the use of a one preemptive dose of mirtazapine versus a one preemptive dose of dexamethasone efficiently decreased the incidence and severity of PSAS in comparison to placebo controls in gynecological procedures under SA. In addition, incidence of hypotensive episodes, pruritus, nausea, and vomiting were lower in M and D groups in comparison to C group.

Both physical and therapeutic strategies have been used to diminish loss of tympanic temperature for prevention of PSAS. In addition, the use of forced-air warming devices and meperidine to maintain tympanic temperatures of patients at ≥36.5°C is also recommended by the ASA guidelines [25]. Nonetheless, potential side effects of meperidine were previously described [26]. So, the investigators conducted this study to possibly seek medications with insignificant adverse effects to substitute the utilization of IV meperidine for management of PSAS.

Maximal effects of the three mechanisms of SA causing core hypothermia occur at the 1^st^ 30–60 min after the subarachnoid block necessitating patients' monitoring, actively warming and antishivering treatment. So, the research team chose the 1^st^ 90 minutes after SA as a time frame for this study [3]. In addition, an anecdotally endorsed dose for oral mirtazapine is a single administration of 30 mg tablet [10, 27] taken 2 hours prior to surgery [27], whereas the selected protocol for dexamethasone administration was based on earlier research [13] and adhered to optimal dose [12–15] for prevention of postoperative nausea and vomiting after laparoscopic cholecystectomy (LC) [15].

Patients' demographic characteristics and patients' perioperative data of the three groups were comparable (Table 1). The investigators of this research reported that M group had a significantly faster regression times by two segments and a significantly shorter duration of analgesia than placebo-treated patients. The investigators suggested an explanation to these findings by the similarity between mirtazapine and granisetron, contrary to ondansetron, that acts on mixed receptors and strongly and selectively binds to the 5-HT_3_ receptors with decreased or no affinity for other 5-HT receptors. Moreover, mirtazapine may affect pain modulation of the spinal cord through antagonism of 5-HT_3_ receptors [28, 29]. Moreover, results of this study matched with previous studies assessing the advantages of dexamethasone whether IV [3, 15, 30] or intrathecally [26] in reducing the time to the highest dermatome block level and increasing both the regression times by two segments and the duration of analgesia.

Results of this study revealed that the incidence of hypotensive episodes after SA during the study period was lower in M group due to the 5-HT_3_ blocking properties of mirtazapine as displayed by other 5-HT_3_ receptor antagonists [29, 31]. Furthermore, a previous study reported about possible mechanisms of dexamethasone in attenuation of PSA hypotension [16]. Terkawi et al., in contrast to our results, reported that ondansetron premedication did not attenuate hemodynamic changes after SA; did not reduce the amount of vasopressor use; and did not decrease the incidence of pruritus, nausea, and vomiting [32].

SA-induced vasodilation in the lower half of body will lead to loss of thermoregulation and core hypothermia whereas vasoconstriction and shivering will be confined to the upper half of the body to augment tympanic temperature [3]. The research team reported a higher incidence of clinically significant shivering in placebo-treated patients in spite of significant differences between basal and 90 minutes after SA tympanic temperature measurements in the three groups following high level of the subarachnoid block [33, 34]. This might be explained by mirtazapine-induced serotonin uptake inhibition in the preoptic anterior hypothalamic part which controls heat production and loss [34]. In addition, anti-inflammatory properties of dexamethasone allow reduction of temperature gradient between tympanic and skin temperatures [12]. Similar to the current study, Shen et al. [35] and a plethora of studies [34, 36] documented that prophylactic 5-HT_3_ receptor antagonists were efficient for decreasing the occurrence of perioperative shivering (POS) in patients after SA. In addition, Kelsaka et al. also reported no significant difference in the incidence of shivering between ondansetron and meperidine groups in orthopedic surgeries under SA [34]. Additionally, previous studies revealed that dexamethasone decreased postanesthetic shivering [12, 15]. Over and above, earlier clinical study had documented equal efficacy of spinal dexamethasone and spinal meperidine for reducing the shivering threshold in comparison to control group in transurethral prostatectomy under SA [26]. Moreover, our results were supported by prior studies regarding high doses of meperidine used to manage PSAS in placebo controls in comparison to intervention groups [22, 36].

The results of Abdel-Ghaffar et al. and Chen et al. were in concordance with our recent findings concerning the lower percentage of pruritus, nausea episodes, and vomiting episodes in M group and they attributed the antipruritic and antiemetic efficacy due to 5-HT_3_ receptor blockers properties of mirtazapine [27, 36]. In addition, 5-HT_3_ antagonists, like ondansetron, and granisetron have been utilized to forestall the neuraxial opioid-induced pruritus. Furthermore, mirtazapine has strong antihistamine effect, exerts its antipruritic effect through activating the *k*-opioid system, and reduces the perception of pruritus through action on the cerebral cortex [37]. In spite of the antiemetic and anti-inflammatory properties of dexamethasone, it lacks an antipruritic effect [38]. However, earlier research showed reduced severity of pruritus in dexamethasone-treated patients compared to placebo-treated patients which reinforced outcomes of this study [39]. Furthermore, the high percentage of pruritus, nausea, and vomiting in C group cases may be due to increased utilization of meperidine [26]. In addition, the research team recorded increased utilization of ephedrine in C group cases which might explain probability of systemic hypersensitivity reactions [40].

Chen et al. recorded that premedication with mirtazapine reduced preoperative anxiety in patients undergoing gynecological operations [27]. Those results were consistent with this study that affirmed the sedative response of mirtazapine as proved by higher Ramsay sedation scores. Moreover, use of dexamethasone improves mood and it could also lead to a greater feeling of well-being due to primary central nervous system impact of steroids [15]. For the aforementioned merits of mirtazapine and dexamethasone, this might justify high satisfaction scores in mirtazapine and dexamethasone groups in comparison to placebo-treated patients.

This clinical trial suffered distinct limits. To begin with, this research needed warmed IV fluids. At our institution, the utilization of warmed IV fluids is held for emergency procedures and for all lengthy procedures. Nevertheless, the research team trusted that the utilization of mirtazapine and dexamethasone in this clinical trial furnished an easy, demonstrated adequacy as antiemetic, less side effects, and more economical for prevention of PSAS in countries with low financial resources. Second, the consequences of this study did exclude endoscopic urosurgical procedures and invasive procedures interconnected with increased blood loss. In spite of that, the investigators proved that mirtazapine and dexamethasone diminished the impact of high levels of intrathecal block which reduced the tympanic temperature threshold for shivering [33, 34]. Furthermore, mirtazapine and dexamethasone showed the favorable feedback to stay away from the techniques used to escape the PSA hypotension (e.g., volume loading or vasopressor use) which might augment the risk of hypervolemia as well as myocardial ischemia [16]. Third, this clinical trial was completed at a single center. Even so, the research team considered that the randomized and the double-blind plan diminished the chance of bias and the comparatively big sample size accomplished significant differences in the side effects that happened. Fourth, the glycemic outline and the percentage of surgical site infections following dexamethasone intake should have been described.

The principle that prevention is better than cure has proven to be true for shivering also and it should be applied. Since the proposed protocol for prevention of PSAS was efficient, easy, more economical, and comparatively free from danger, the investigators recommend the utilization of mirtazapine and dexamethasone for prevention of PSAS in patients subjected to risk factors [33].

## 6. Conclusion

Prophylactic administration of mirtazapine or dexamethasone attenuated shivering with minimal hazards in patients scheduled for gynecological surgeries under spinal anesthesia with priority to mirtazapine.

## Figures and Tables

**Table 1:** Patients' demographics and perioperative data.

Items	M group (*n* = 100)	D group (*n* = 100)	C group (*n* = 100)	*P* value
Age (year)	44.3 ± 7.9	44.8 ± 7.4	42.8 ± 7.2	^^^0.140
BMI (kg/m^2^)	30.4 ± 1.5	30.5 ± 1.6	30.2 ± 1.7	^^^0.288
ASA (I/II)	26/74	27/73	29/71	^#^0.889
Dose of bupivacaine (mg)	14.9 ± 1.9	15.1 ± 1.9	15.4 ± 2.1	^^^0.199
Operation time (min)	113.9 ± 14.4	113.6 ± 13.6	112.5 ± 13.7	^^^0.761

*Types of operations; n, %*				
(i) Total abdominal hysterectomy (TAH)	14 (14%)	13 (13%)	12 (12%)	^#^0.998
(ii) TAH + BSO	40 (40%)	41 (41%)	39 (39%)	
(iii) Vaginal hysterectomy (VH)	9 (9%)	10 (10%)	8 (8%)	
(iv) VH + PFR	27 (27%)	26 (26%)	28 (28%)	
(v) Vesicovaginal fistula repair	10 (10%)	10 (10%)	13 (13%)	
Preoperative Hb (g/dl)	11.5 ± 0.4	11.5 ± 0.4	11.6 ± 0.4	^^^0.141
Postoperative Hb (g/dl)	9.3 ± 0.5	9.3 ± 0.4	9.4 ± 0.4	^^^0.342
Total IV fluids used (ml)	2035.0 ± 111.4	2007.0 ± 117.4	2023.0 ± 116.2	^^^0.227

*Characteristics of neuraxial anesthesia techniques*				
Peak sensory level	T5 (T4–T8)	T6 (T4–T8)	T6 (T4–T8)	^§^0.389
Time to peak sensory level (min)	6.5 ± 0.6a	4.6 ± 0.6b	6.7 ± 0.6a	^^^<0.001^*∗*^
Time to two-segment regression (min)	63.2 ± 1.7a	77.1 ± 1.4b	69.3 ± 1.2c	^^^<0.001^*∗*^
Time to reach maximum motor block (min)	9.2 ± 0.4	9.1 ± 0.5	9.2 ± 0.4	^^^0.062
Duration of motor block (min)	135.2 ± 2.4	135.9 ± 2.2	135.4 ± 2.3	^^^0.065
Time to rescue analgesia (min)	173.9 ± 4.1a	321.3 ± 4.8 b	215.8 ± 4.4c	^^^<0.001^*∗*^

Data were presented as median (range), mean (SD), numbers, and percent. ^^^ANOVA test, ^#^chi square test, and ^§^Kruskal Wallis test. Labels (a, b, c) denote homogenous groups depending on post hoc Bonferroni test. ^*∗*^Statistically significant. TAH + BSO: total abdominal hysterectomy with a bilateral salpingo-oophorectomy. PFR: pelvic floor repair. M group: mirtazapine group; D group: dexamethasone group; C group: control group.

**Table 2:** Incidence, grades, and treatment of postspinal anesthesia shivering among the studied groups.

Time points	M group (*n* = 100)	D group (*n* = 100)	C group (*n* = 100)	*P* value
Incidence of shivering; *n*, %	41 (41%) a	62 (62%) b	91 (91%) c	^#^<0.001^*∗*^
*Grade; n, %*				
(i) 0	59 (59%) a	38 (38%) b	9 (9%) c	^#^<0.001^*∗*^
(ii) I	25(25%) a	31 (31%) a	17 (17%) a	
(iii) II	10 (10%) a	19 (19%) a	43 (43%) b	
(iv) III	6 (6%) a	12 (12%) a	31 (31%) b	
Patients with clinically significant shivering (Grade ≥2); *n*, %	16 (16%) a	31 (31%) b	74 (74%) c	^#^<0.001^*∗*^
Onset of shivering (min)	51.3 ± 5.2 a	32.8 ± 3.5 b	16.0 ± 5.4 c	^^^<0.001^*∗*^
Dose of meperidine (mg)	25.1 ± 1.2 a	31.3 ± 4.7b	36.1 ± 3.5 c	^^^ **<0.001** ^*∗*^
Response rate after administration of 1^st^ dose of meperidine; *n*, %	16 (100%) a	22 (71%) b	41 (55.4%) b	^**#**^ **0.002** ^*∗*^
Recurrence; *n*, %	0 (0.0%) a	9 (29%) b	33 (44.6%) b	^**#**^ **0.002** ^*∗*^

Data were presented as numbers and percent. ^#^Chi square test and ^^^ANOVA test. Labels (a, b, c) denote homogenous groups depending on post hoc Bonferroni test. ^*∗*^Statistically significant. M group: mirtazapine group; D group: dexamethasone group; C group: control group.

**Table 3:** Side effects, administered treatments, and patient satisfaction score.

Time points	M group (*n* = 100)	D group (*n* = 100)	C group (*n* = 100)	*P* value
Bradycardia; *n*,%	6 (6%)	8 (8%)	7 (7%)	^#^0.858
Hypotension; *n*,%	6 (6%) a	8 (8%) a	25 (25%) b	^#^<0.001^*∗*^
Need for ephedrine; *n*, %	27 (27%) a	32 (32%) a	78 (78%) b	^#^<0.001^*∗*^
Ephedrine dose (mg)	10.3 ± 3.1 a	13.5 ± 4 a	21.4 ± 6.9 b	^**^**^ **<0.001** ^*∗*^
Pruritus; *n*, %	2 (2%) a	10 (10%) a	24 (24%) b	^#^<0.001^*∗*^
Nausea; *n*, %	4 (4%) a	5 (5%) a	28 (28%) b	^#^<0.001^*∗*^
Vomiting; *n*, %	2 (2%) a	3 (3%) a	17 (17%) b	^#^<0.001^*∗*^
Rescue antiemetic; *n*, %	3 (3%) a	4 (4%) a	22 (22%) b	^#^<0.001^*∗*^
*Sedation; n, %*				
(i) I	7 (7%) a	100 (100%) b	100 (100%)b	^#^<0.001^*∗*^
(ii) II	85 (85%)	0 (0)	0 (0)	
(iii) III	8 (8%)	0 (0)	0 (0)	
(iv) IV	0 (0)	0 (0)	0 (0)	
Headache; *n*, %	6 (6%)	4 (4%)	5 (5%)	^#^0.810
Dry mouth; *n*, %	22 (22%) a	8 (8%) b	10 (10%) b	^**#**^ **0.007** ^*∗*^
Elevated liver enzymes; *n*, %	3 (3%)	1 (1%)	1 (1%)	^⌂^0.625
Patient satisfaction score	5.0 (5-6) a	5.0 (4–6) a	2.0 (2-3) b	^§^<0.001^*∗*^

Data were presented as median (range), mean (SD), numbers, and percent. ^#^Chi square test, ^^^ANOVA test, ^§^Kruskal Wallis test, and ^⌂^Fisher's exact test. Labels (a, b, c) denote homogenous groups depending on post hoc Bonferroni test. ^*∗*^Statistically significant. M group: mirtazapine group; D group: dexamethasone group; C group: control group.

## Data Availability

The datasets generated and analyzed during the present study are available from the corresponding author on reasonable request.
